# P-1308. Survey of U.S. Hunters: Behaviors that May Increase Exposure to Chronic Wasting Disease

**DOI:** 10.1093/ofid/ofae631.1489

**Published:** 2025-01-29

**Authors:** Colton P Boney, Connor R Lindenmuth, Nicholas J Trapp, Kevin J Milbert, Tony L Moore, Robert W Parker

**Affiliations:** The Alabama College of Osteopathic Medicine, Huntsville, Alabama; Alabama College of Osteopathic Medicine, Panama City, Florida; Alabama College of Osteopathic, ENTERPRISE, Alabama; ACOM, Lake Worth, Florida; University of New England, Apex, North Carolina; Alabama College of Osteopathic Medicine, Panama City, Florida

## Abstract

**Background:**

Chronic Wasting Disease (CWD) is caused by a prion leading to transmissible spongiform encephalopathy primarily in members of the *Cervidae* family such as deer, elk, and moose. At this time, there is insufficient data to suggest possible zoonosis. Although there have been no recorded cases of CWD in humans, studies have shown that squirrel monkeys (*Saimiri sciureus*) are susceptible to transmission of CWD in a laboratory setting. The goal of this survey was to collect and examine data about safe handling practices, awareness of CWD, testing use, and meat consumption among United States hunters.

**Methods:**

The survey included questions regarding basic demographics, butchering, taxidermy, awareness of CWD, testing frequency/behaviors, and barriers to testing. Participants were recruited through state organizations as well as hunting/outdoors-associated social media pages. Hunters of deer, elk, or moose were invited to participate in the study if they had harvested one of those game species in the past five years. States were grouped in accordance with CDC-described regions to compare for statistical trends among the data.

**Results:**

125 individuals participated in the survey. The majority consumed meat from all (82%) or most (9.6%) of their game. Respondents commonly tested none (67%) or some (20%) of the meat they consumed. Though 33.1% tested their harvest for CWD, 58% of those who tested did not wait for CWD test results before consuming the meat. In addition to meat consumption, 66% used meat processing plants, 86% participated in field dressing, and 41% taxidermized their animals at home. Barriers to testing included the belief that CWD was not in their respective area (44%) or that they did not have access to testing equipment and/or other resources (39%).

**Conclusion:**

The survey highlighted that hunters who frequently consume meat from members of the *Cervidae* family in states with CWD are not frequently testing due to poor accessibility and lack of data monitoring endemic spread. This may lead to increased exposure risk for those unaware of the illness or believe that CWD is not in their game populations. More data is needed to understand the full extent of exposure risk as transmission risk of CWD to humans is further researched.

**Disclosures:**

**All Authors**: No reported disclosures

